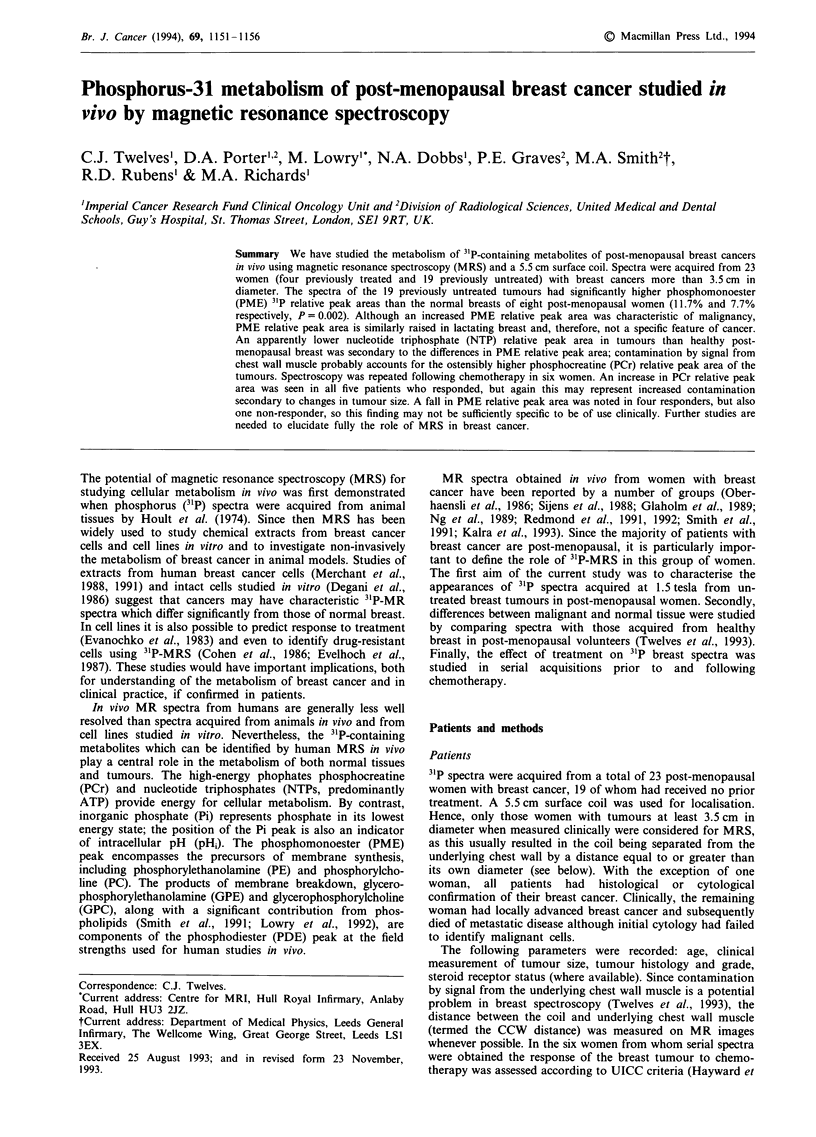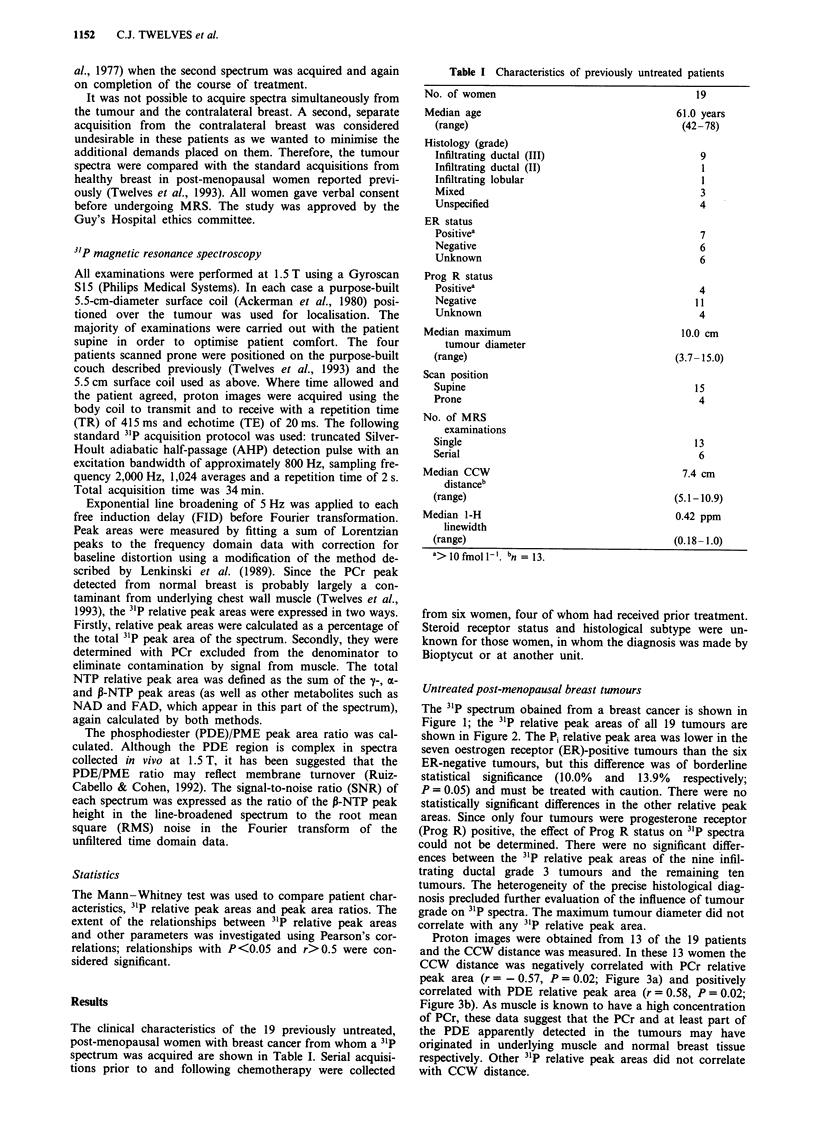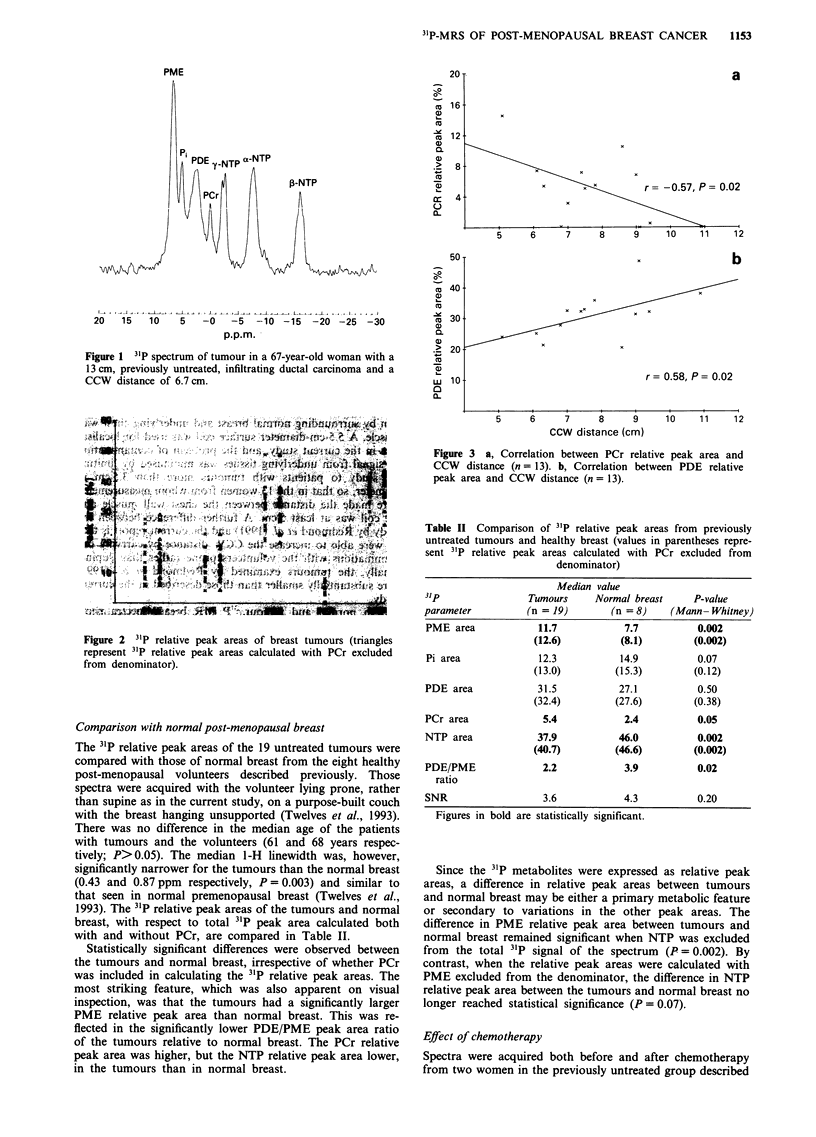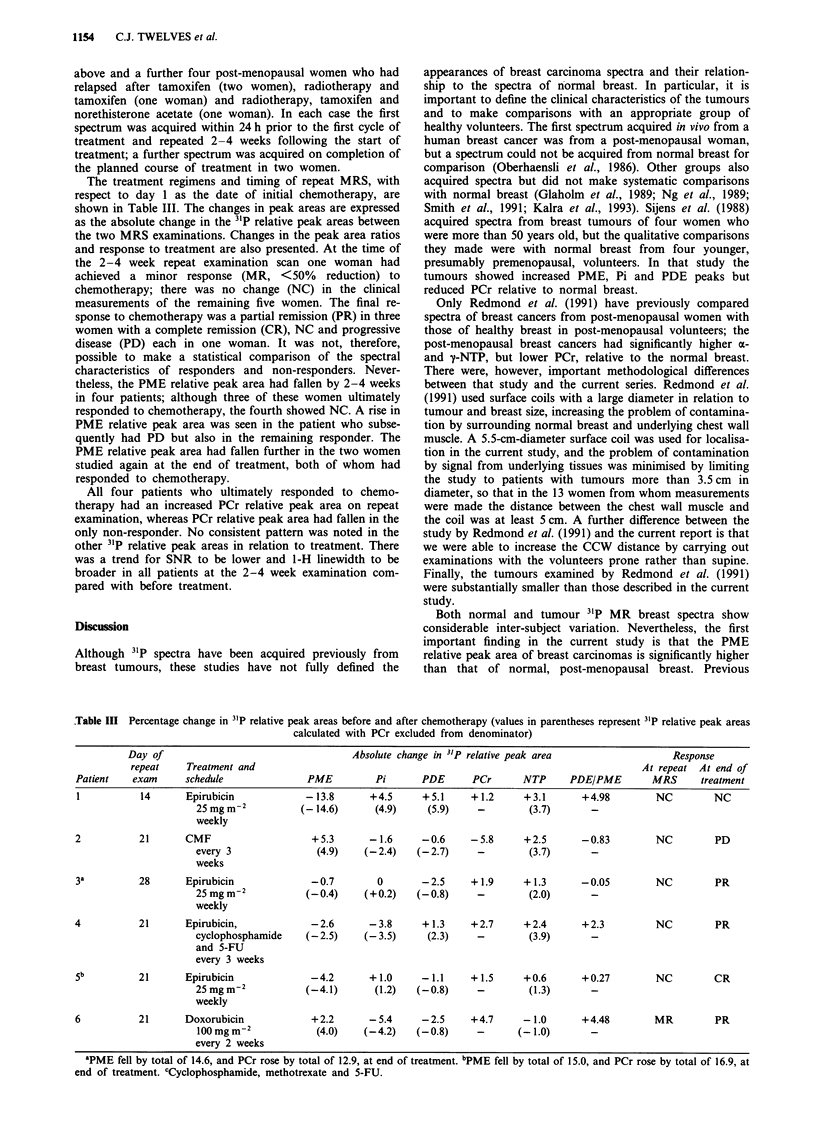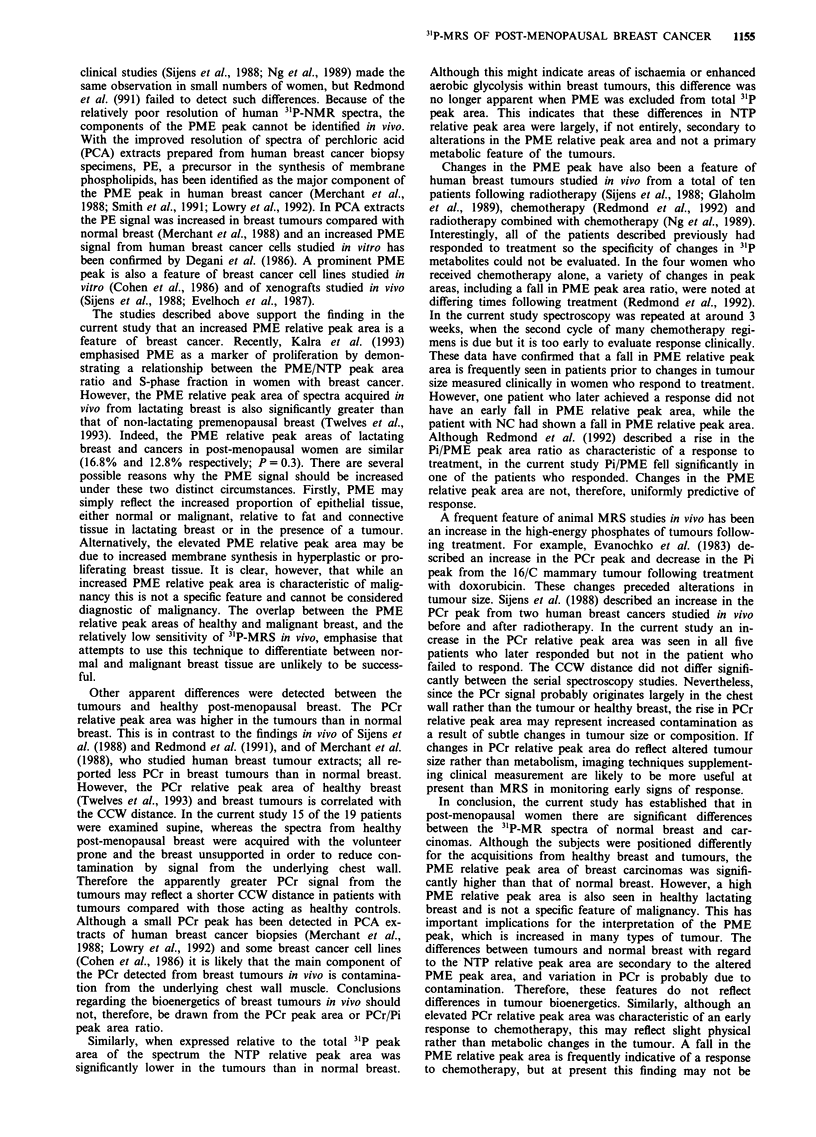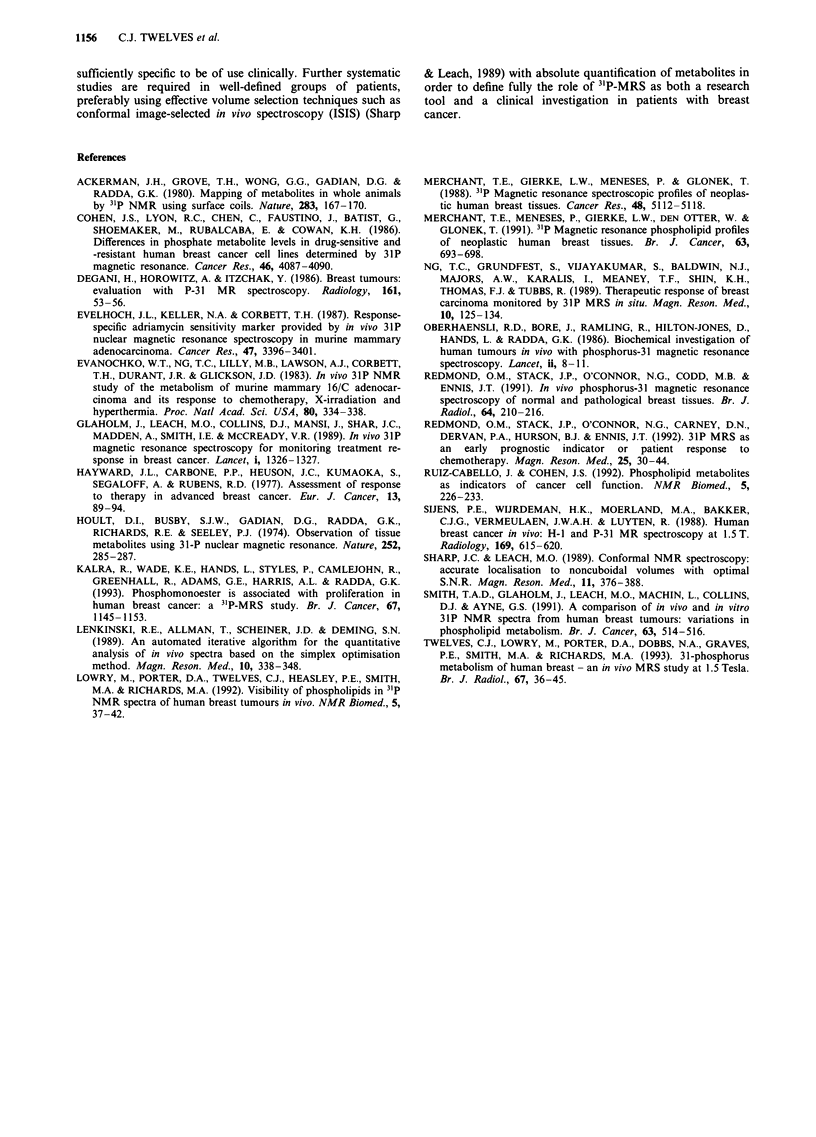# Phosphorus-31 metabolism of post-menopausal breast cancer studied in vivo by magnetic resonance spectroscopy.

**DOI:** 10.1038/bjc.1994.226

**Published:** 1994-06

**Authors:** C. J. Twelves, D. A. Porter, M. Lowry, N. A. Dobbs, P. E. Graves, M. A. Smith, R. D. Rubens, M. A. Richards

**Affiliations:** Imperial Cancer Research Fund Clinical Oncology Unit, United Medical School, Guy's Hospital, London, UK.

## Abstract

We have studied the metabolism of 31P-containing metabolites of post-menopausal breast cancers in vivo using magnetic resonance spectroscopy (MRS) and a 5.5 cm surface coil. Spectra were acquired from 23 diameter. The spectra of the 19 previously untreated tumours had significantly higher phosphomonoester (PME) 31P relative peak areas than the normal breasts of eight post-menopausal women (11.7% and 7.7% respectively, P = 0.002). Although an increased PME relative peak area was characteristic of malignancy, PME relative peak area is similarly raised in lactating breast and, therefore, not a specific feature of cancer. An apparently lower nucleotide triphosphate (NTP) relative peak area in tumours than healthy postmenopausal breast was secondary to the differences in PME relative peak area; contamination by signal from chest wall muscle probably accounts for the ostensibly higher phosphocreatine (PCr) relative peak area of the tumours. Spectroscopy was repeated following chemotherapy in six women. An increase in PCr relative peak area was seen in all five patients who responded, but again this may represent increased contamination secondary to changes in tumour size. A fall in PME relative peak area was noted in four responders, but also one non-responder, so this finding may not be sufficiently specific to be of use clinically. Further studies are need to elucidate fully the role of MRS in breast cancer.


 © Macmillan Press Ltd., 1994

### Phosphorus-31 metabolism of post-menopausal breast cancer studied *in vivo* by magnetic resonance spectroscopy

C.J. Twelves[1], D.A. Porter[1,2], M. Lowry[1*], N.A. Dobbs[1], P.E. Graves[2], M.A. Smith[2†], R.D. Rubens[1] & M.A. Richards[1]

[1]*Imperial Cancer Research Fund Clinical Oncology Unit and* [2]*Division of Radiological Sciences, United Medical and Dental Schools, Guy's Hospital, St. Thomas Street, London, SE1 9RT, UK.*

**Summary** We have studied the metabolism of $^{31}$P-containing metabolites of post-menopausal breast cancers *in vivo* using magnetic resonance spectroscopy (MRS) and a 5.5 cm surface coil. Spectra were acquired from 23 women (four previously treated and 19 previously untreated) with breast cancers more than 3.5 cm in diameter. The spectra of the 19 previously untreated tumours had significantly higher phosphomonoester (PME) $^{31}$P relative peak areas than the normal breasts of eight post-menopausal women (11.7% and 7.7% respectively, $P = 0.002$). Although an increased PME relative peak area was characteristic of malignancy, PME relative peak area is similarly raised in lactating breast and, therefore, not a specific feature of cancer. An apparently lower nucleotide triphosphate (NTP) relative peak area in tumours than healthy post-menopausal breast was secondary to the differences in PME relative peak area; contamination by signal from chest wall muscle probably accounts for the ostensibly higher phosphocreatine (PCr) relative peak area of the tumours. Spectroscopy was repeated following chemotherapy in six women. An increase in PCr relative peak area was seen in all five patients who responded, but again this may represent increased contamination secondary to changes in tumour size. A fall in PME relative peak area was noted in four responders, but also one non-responder, so this finding may not be sufficiently specific to be of use clinically. Further studies are needed to elucidate fully the role of MRS in breast cancer.

The potential of magnetic resonance spectroscopy (MRS) for studying cellular metabolism *in vivo* was first demonstrated when phosphorus ($^{31}$P) spectra were acquired from animal tissues by Hoult *et al.* (1974). Since then MRS has been widely used to study chemical extracts from breast cancer cells and cell lines *in vitro* and to investigate non-invasively the metabolism of breast cancer in animal models. Studies of extracts from human breast cancer cells (Merchant *et al.*, 1988, 1991) and intact cells studied *in vitro* (Degani *et al.*, 1986) suggest that cancers may have characteristic $^{31}$P-MR spectra which differ significantly from those of normal breast. In cell lines it is also possible to predict response to treatment (Evanochko *et al.*, 1983) and even to identify drug-resistant cells using $^{31}$P-MRS (Cohen *et al.*, 1986; Evelhoch *et al.*, 1987). These studies would have important implications, both for understanding of the metabolism of breast cancer and in clinical practice, if confirmed in patients.

*In vivo* MR spectra from humans are generally less well resolved than spectra acquired from animals *in vivo* and from cell lines studied *in vitro*. Nevertheless, the $^{31}$P-containing metabolites which can be identified by human MRS *in vivo* play a central role in the metabolism of both normal tissues and tumours. The high-energy phophates phosphocreatine (PCr) and nucleotide triphosphates (NTPs, predominantly ATP) provide energy for cellular metabolism. By contrast, inorganic phosphate (Pi) represents phosphate in its lowest energy state; the position of the Pi peak is also an indicator of intracellular pH ($pH_i$). The phosphomonoester (PME) peak encompasses the precursors of membrane synthesis, including phosphorylethanolamine (PE) and phosphorylcholine (PC). The products of membrane breakdown, glycerophosphorylethanolamine (GPE) and glycerophosphorylcholine (GPC), along with a significant contribution from phospholipids (Smith *et al.*, 1991; Lowry *et al.*, 1992), are components of the phosphodiester (PDE) peak at the field strengths used for human studies *in vivo*.

Correspondence: C.J. Twelves.
*Current address: Centre for MRI, Hull Royal Infirmary, Anlaby Road, Hull HU3 2JZ.
†Current address: Department of Medical Physics, Leeds General Infirmary, The Wellcome Wing, Great George Street, Leeds LS1 3EX.
Received 25 August 1993; and in revised form 23 November, 1993.

MR spectra obtained *in vivo* from women with breast cancer have been reported by a number of groups (Oberhaensli *et al.*, 1986; Sijens *et al.*, 1988; Glaholm *et al.*, 1989; Ng *et al.*, 1989; Redmond *et al.*, 1991, 1992; Smith *et al.*, 1991; Kalra *et al.*, 1993). Since the majority of patients with breast cancer are post-menopausal, it is particularly important to define the role of $^{31}$P-MRS in this group of women. The first aim of the current study was to characterise the appearances of $^{31}$P spectra acquired at 1.5 tesla from untreated breast tumours in post-menopausal women. Secondly, differences between malignant and normal tissue were studied by comparing spectra with those acquired from healthy breast in post-menopausal volunteers (Twelves *et al.*, 1993). Finally, the effect of treatment on $^{31}$P breast spectra was studied in serial acquisitions prior to and following chemotherapy.

### Patients and methods

### *Patients*

$^{31}$P spectra were acquired from a total of 23 post-menopausal women with breast cancer, 19 of whom had received no prior treatment. A 5.5 cm surface coil was used for localisation. Hence, only those women with tumours at least 3.5 cm in diameter when measured clinically were considered for MRS, as this usually resulted in the coil being separated from the underlying chest wall by a distance equal to or greater than its own diameter (see below). With the exception of one woman, all patients had histological or cytological confirmation of their breast cancer. Clinically, the remaining woman had locally advanced breast cancer and subsequently died of metastatic disease although initial cytology had failed to identify malignant cells.

The following parameters were recorded: age, clinical measurement of tumour size, tumour histology and grade, steroid receptor status (where available). Since contamination by signal from the underlying chest wall muscle is a potential problem in breast spectroscopy (Twelves *et al.*, 1993), the distance between the coil and underlying chest wall muscle (termed the CCW distance) was measured on MR images whenever possible. In the six women from whom serial spectra were obtained the response of the breast tumour to chemotherapy was assessed according to UICC criteria (Hayward *et*

al., 1977) when the second spectrum was acquired and again on completion of the course of treatment.

It was not possible to acquire spectra simultaneously from the tumour and the contralateral breast. A second, separate acquisition from the contralateral breast was considered undesirable in these patients as we wanted to minimise the additional demands placed on them. Therefore, the tumour spectra were compared with the standard acquisitions from healthy breast in post-menopausal women reported previously (Twelves *et al.*, 1993). All women gave verbal consent before undergoing MRS. The study was approved by the Guy's Hospital ethics committee.

### *$^{31}P$ magnetic resonance spectroscopy*

All examinations were performed at 1.5 T using a Gyroscan S15 (Philips Medical Systems). In each case a purpose-built 5.5-cm-diameter surface coil (Ackerman *et al.*, 1980) positioned over the tumour was used for localisation. The majority of examinations were carried out with the patient supine in order to optimise patient comfort. The four patients scanned prone were positioned on the purpose-built couch described previously (Twelves *et al.*, 1993) and the 5.5 cm surface coil used as above. Where time allowed and the patient agreed, proton images were acquired using the body coil to transmit and to receive with a repetition time (TR) of 415 ms and echotime (TE) of 20 ms. The following standard $^{31}P$ acquisition protocol was used: truncated Silver-Hoult adiabatic half-passage (AHP) detection pulse with an excitation bandwidth of approximately 800 Hz, sampling frequency 2,000 Hz, 1,024 averages and a repetition time of 2 s. Total acquisition time was 34 min.

Exponential line broadening of 5 Hz was applied to each free induction delay (FID) before Fourier transformation. Peak areas were measured by fitting a sum of Lorentzian peaks to the frequency domain data with correction for baseline distortion using a modification of the method described by Lenkinski *et al.* (1989). Since the PCr peak detected from normal breast is probably largely a contaminant from underlying chest wall muscle (Twelves *et al.*, 1993), the $^{31}P$ relative peak areas were expressed in two ways. Firstly, relative peak areas were calculated as a percentage of the total $^{31}P$ peak area of the spectrum. Secondly, they were determined with PCr excluded from the denominator to eliminate contamination by signal from muscle. The total NTP relative peak area was defined as the sum of the $\gamma$-, $\alpha$- and $\beta$-NTP peak areas (as well as other metabolites such as NAD and FAD, which appear in this part of the spectrum), again calculated by both methods.

The phosphodiester (PDE)/PME peak area ratio was calculated. Although the PDE region is complex in spectra collected *in vivo* at 1.5 T, it has been suggested that the PDE/PME ratio may reflect membrane turnover (Ruiz-Cabello & Cohen, 1992). The signal-to-noise ratio (SNR) of each spectrum was expressed as the ratio of the $\beta$-NTP peak height in the line-broadened spectrum to the root mean square (RMS) noise in the Fourier transform of the unfiltered time domain data.

### *Statistics*

The Mann–Whitney test was used to compare patient characteristics, $^{31}P$ relative peak areas and peak area ratios. The extent of the relationships between $^{31}P$ relative peak areas and other parameters was investigated using Pearson's correlations; relationships with $P<0.05$ and $r>0.5$ were considered significant.

### Results

The clinical characteristics of the 19 previously untreated, post-menopausal women with breast cancer from whom a $^{31}P$ spectrum was acquired are shown in Table I. Serial acquisitions prior to and following chemotherapy were collected from six women, four of whom had received prior treatment. Steroid receptor status and histological subtype were unknown for those women, in whom the diagnosis was made by Bioptycut or at another unit.

**Table I** Characteristics of previously untreated patients

| | |
|---|---|
| No. of women | 19 |
| Median age | 61.0 years |
| (range) | (42–78) |
| Histology (grade) | |
| Infiltrating ductal (III) | 9 |
| Infiltrating ductal (II) | 1 |
| Infiltrating lobular | 1 |
| Mixed | 3 |
| Unspecified | 4 |
| ER status | |
| Positive[a] | 7 |
| Negative | 6 |
| Unknown | 6 |
| Prog R status | |
| Positive[a] | 4 |
| Negative | 11 |
| Unknown | 4 |
| Median maximum tumour diameter | 10.0 cm |
| (range) | (3.7–15.0) |
| Scan position | |
| Supine | 15 |
| Prone | 4 |
| No. of MRS examinations | |
| Single | 13 |
| Serial | 6 |
| Median CCW distance[b] | 7.4 cm |
| (range) | (5.1–10.9) |
| Median 1-H linewidth | 0.42 ppm |
| (range) | (0.18–1.0) |

[a]$>10\ \mathrm{fmol\ l^{-1}}$. [b]$n = 13$.

### *Untreated post-menopausal breast tumours*

The $^{31}P$ spectrum obained from a breast cancer is shown in Figure 1; the $^{31}P$ relative peak areas of all 19 tumours are shown in Figure 2. The $P_i$ relative peak area was lower in the seven oestrogen receptor (ER)-positive tumours than the six ER-negative tumours, but this difference was of borderline statistical significance (10.0% and 13.9% respectively; $P=0.05$) and must be treated with caution. There were no statistically significant differences in the other relative peak areas. Since only four tumours were progesterone receptor (Prog R) positive, the effect of Prog R status on $^{31}P$ spectra could not be determined. There were no significant differences between the $^{31}P$ relative peak areas of the nine infiltrating ductal grade 3 tumours and the remaining ten tumours. The heterogeneity of the precise histological diagnosis precluded further evaluation of the influence of tumour grade on $^{31}P$ spectra. The maximum tumour diameter did not correlate with any $^{31}P$ relative peak area.

Proton images were obtained from 13 of the 19 patients and the CCW distance was measured. In these 13 women the CCW distance was negatively correlated with PCr relative peak area ($r=-0.57$, $P=0.02$; Figure 3a) and positively correlated with PDE relative peak area ($r=0.58$, $P=0.02$; Figure 3b). As muscle is known to have a high concentration of PCr, these data suggest that the PCr and at least part of the PDE apparently detected in the tumours may have originated in underlying muscle and normal breast tissue respectively. Other $^{31}P$ relative peak areas did not correlate with CCW distance.

**Figure 1** $^{31}$P spectrum of tumour in a 67-year-old woman with a 13 cm, previously untreated, infiltrating ductal carcinoma and a CCW distance of 6.7 cm.

**Figure 2** $^{31}$P relative peak areas of breast tumours (triangles represent $^{31}$P relative peak areas calculated with PCr excluded from denominator).

### Comparison with normal post-menopausal breast

The $^{31}$P relative peak areas of the 19 untreated tumours were compared with those of normal breast from the eight healthy post-menopausal volunteers described previously. Those spectra were acquired with the volunteer lying prone, rather than supine as in the current study, on a purpose-built couch with the breast hanging unsupported (Twelves *et al.*, 1993). There was no difference in the median age of the patients with tumours and the volunteers (61 and 68 years respectively; $P > 0.05$). The median 1-H linewidth was, however, significantly narrower for the tumours than the normal breast (0.43 and 0.87 ppm respectively, $P = 0.003$) and similar to that seen in normal premenopausal breast (Twelves *et al.*, 1993). The $^{31}$P relative peak areas of the tumours and normal breast, with respect to total $^{31}$P peak area calculated both with and without PCr, are compared in Table II.

Statistically significant differences were observed between the tumours and normal breast, irrespective of whether PCr was included in calculating the $^{31}$P relative peak areas. The most striking feature, which was also apparent on visual inspection, was that the tumours had a significantly larger PME relative peak area than normal breast. This was reflected in the significantly lower PDE/PME peak area ratio of the tumours relative to normal breast. The PCr relative peak area was higher, but the NTP relative peak area lower, in the tumours than in normal breast.

**Figure 3** **a**, Correlation between PCr relative peak area and CCW distance ($n = 13$). **b**, Correlation between PDE relative peak area and CCW distance ($n = 13$).

**Table II** Comparison of $^{31}$P relative peak areas from previously untreated tumours and healthy breast (values in parentheses represent $^{31}$P relative peak areas calculated with PCr excluded from denominator)

| $^{31}P$ parameter | Median value Tumours (n = 19) | Median value Normal breast (n = 8) | P-value (Mann–Whitney) |
|---|---|---|---|
| PME area | **11.7** **(12.6)** | **7.7** **(8.1)** | **0.002** **(0.002)** |
| Pi area | 12.3 (13.0) | 14.9 (15.3) | 0.07 (0.12) |
| PDE area | 31.5 (32.4) | 27.1 (27.6) | 0.50 (0.38) |
| PCr area | **5.4** | **2.4** | **0.05** |
| NTP area | **37.9** **(40.7)** | **46.0** **(46.6)** | **0.002** **(0.002)** |
| PDE/PME ratio | **2.2** | **3.9** | **0.02** |
| SNR | 3.6 | 4.3 | 0.20 |

Figures in bold are statistically significant.

Since the $^{31}$P metabolites were expressed as relative peak areas, a difference in relative peak areas between tumours and normal breast may be either a primary metabolic feature or secondary to variations in the other peak areas. The difference in PME relative peak area between tumours and normal breast remained significant when NTP was excluded from the total $^{31}$P signal of the spectrum ($P = 0.002$). By contrast, when the relative peak areas were calculated with PME excluded from the denominator, the difference in NTP relative peak area between the tumours and normal breast no longer reached statistical significance ($P = 0.07$).

### Effect of chemotherapy

Spectra were acquired both before and after chemotherapy from two women in the previously untreated group described above and a further four post-menopausal women who had relapsed after tamoxifen (two women), radiotherapy and tamoxifen (one woman) and radiotherapy, tamoxifen and norethisterone acetate (one woman). In each case the first spectrum was acquired within 24 h prior to the first cycle of treatment and repeated 2–4 weeks following the start of treatment; a further spectrum was acquired on completion of the planned course of treatment in two women.

The treatment regimens and timing of repeat MRS, with respect to day 1 as the date of initial chemotherapy, are shown in Table III. The changes in peak areas are expressed as the absolute change in the $^{31}$P relative peak areas between the two MRS examinations. Changes in the peak area ratios and response to treatment are also presented. At the time of the 2–4 week repeat examination scan one woman had achieved a minor response (MR, <50% reduction) to chemotherapy; there was no change (NC) in the clinical measurements of the remaining five women. The final response to chemotherapy was a partial remission (PR) in three women with a complete remission (CR), NC and progressive disease (PD) each in one woman. It was not, therefore, possible to make a statistical comparison of the spectral characteristics of responders and non-responders. Nevertheless, the PME relative peak area had fallen by 2–4 weeks in four patients; although three of these women ultimately responded to chemotherapy, the fourth showed NC. A rise in PME relative peak area was seen in the patient who subsequently had PD but also in the remaining responder. The PME relative peak area had fallen further in the two women studied again at the end of treatment, both of whom had responded to chemotherapy.

All four patients who ultimately responded to chemotherapy had an increased PCr relative peak area on repeat examination, whereas PCr relative peak area had fallen in the only non-responder. No consistent pattern was noted in the other $^{31}$P relative peak areas in relation to treatment. There was a trend for SNR to be lower and 1-H linewidth to be broader in all patients at the 2–4 week examination compared with before treatment.

### Discussion

Although $^{31}$P spectra have been acquired previously from breast tumours, these studies have not fully defined the appearances of breast carcinoma spectra and their relationship to the spectra of normal breast. In particular, it is important to define the clinical characteristics of the tumours and to make comparisons with an appropriate group of healthy volunteers. The first spectrum acquired *in vivo* from a human breast cancer was from a post-menopausal woman, but a spectrum could not be acquired from normal breast for comparison (Oberhaensli *et al.*, 1986). Other groups also acquired spectra but did not make systematic comparisons with normal breast (Glaholm *et al.*, 1989; Ng *et al.*, 1989; Smith *et al.*, 1991; Kalra *et al.*, 1993). Sijens *et al.* (1988) acquired spectra from breast tumours of four women who were more than 50 years old, but the qualitative comparisons they made were with normal breast from four younger, presumably premenopausal, volunteers. In that study the tumours showed increased PME, Pi and PDE peaks but reduced PCr relative to normal breast.

Only Redmond *et al.* (1991) have previously compared spectra of breast cancers from post-menopausal women with those of healthy breast in post-menopausal volunteers; the post-menopausal breast cancers had significantly higher α- and γ-NTP, but lower PCr, relative to the normal breast. There were, however, important methodological differences between that study and the current series. Redmond *et al.* (1991) used surface coils with a large diameter in relation to tumour and breast size, increasing the problem of contamination by surrounding normal breast and underlying chest wall muscle. A 5.5-cm-diameter surface coil was used for localisation in the current study, and the problem of contamination by signal from underlying tissues was minimised by limiting the study to patients with tumours more than 3.5 cm in diameter, so that in the 13 women from whom measurements were made the distance between the chest wall muscle and the coil was at least 5 cm. A further difference between the study by Redmond *et al.* (1991) and the current report is that we were able to increase the CCW distance by carrying out examinations with the volunteers prone rather than supine. Finally, the tumours examined by Redmond *et al.* (1991) were substantially smaller than those described in the current study.

Both normal and tumour $^{31}$P MR breast spectra show considerable inter-subject variation. Nevertheless, the first important finding in the current study is that the PME relative peak area of breast carcinomas is significantly higher than that of normal, post-menopausal breast. Previous

**Table III** Percentage change in $^{31}$P relative peak areas before and after chemotherapy (values in parentheses represent $^{31}$P relative peak areas calculated with PCr excluded from denominator)

| Patient | Day of repeat exam | Treatment and schedule | Absolute change in $^{31}P$ relative peak area: PME | Pi | PDE | PCr | NTP | PDE/PME | Response: At repeat MRS | At end of treatment |
|---|---|---|---|---|---|---|---|---|---|---|
| 1 | 14 | Epirubicin 25 mg m$^{-2}$ weekly | −13.8 (−14.6) | +4.5 (4.9) | +5.1 (5.9) | +1.2 – | +3.1 (3.7) | +4.98 – | NC | NC |
| 2 | 21 | CMF every 3 weeks | +5.3 (4.9) | −1.6 (−2.4) | −0.6 (−2.7) | −5.8 – | +2.5 (3.7) | −0.83 – | NC | PD |
| 3[a] | 28 | Epirubicin 25 mg m$^{-2}$ weekly | −0.7 (−0.4) | 0 (+0.2) | −2.5 (−0.8) | +1.9 – | +1.3 (2.0) | −0.05 – | NC | PR |
| 4 | 21 | Epirubicin, cyclophosphamide and 5-FU every 3 weeks | −2.6 (−2.5) | −3.8 (−3.5) | +1.3 (2.3) | +2.7 – | +2.4 (3.9) | +2.3 – | NC | PR |
| 5[b] | 21 | Epirubicin 25 mg m$^{-2}$ weekly | −4.2 (−4.1) | +1.0 (1.2) | −1.1 (−0.8) | +1.5 – | +0.6 (1.3) | +0.27 – | NC | CR |
| 6 | 21 | Doxorubicin 100 mg m$^{-2}$ every 2 weeks | +2.2 (4.0) | −5.4 (−4.2) | −2.5 (−0.8) | +4.7 – | −1.0 (−1.0) | +4.48 – | MR | PR |

[a]PME fell by total of 14.6, and PCr rose by total of 12.9, at end of treatment. [b]PME fell by total of 15.0, and PCr rose by total of 16.9, at end of treatment. [c]Cyclophosphamide, methotrexate and 5-FU.

clinical studies (Sijens *et al.*, 1988; Ng *et al.*, 1989) made the same observation in small numbers of women, but Redmond *et al.* (991) failed to detect such differences. Because of the relatively poor resolution of human $^{31}$P-NMR spectra, the components of the PME peak cannot be identified *in vivo*. With the improved resolution of spectra of perchloric acid (PCA) extracts prepared from human breast cancer biopsy specimens, PE, a precursor in the synthesis of membrane phospholipids, has been identified as the major component of the PME peak in human breast cancer (Merchant *et al.*, 1988; Smith *et al.*, 1991; Lowry *et al.*, 1992). In PCA extracts the PE signal was increased in breast tumours compared with normal breast (Merchant *et al.*, 1988) and an increased PME signal from human breast cancer cells studied *in vitro* has been confirmed by Degani *et al.* (1986). A prominent PME peak is also a feature of breast cancer cell lines studied *in vitro* (Cohen *et al.*, 1986) and of xenografts studied *in vivo* (Sijens *et al.*, 1988; Evelhoch *et al.*, 1987).

The studies described above support the finding in the current study that an increased PME relative peak area is a feature of breast cancer. Recently, Kalra *et al.* (1993) emphasised PME as a marker of proliferation by demonstrating a relationship between the PME/NTP peak area ratio and S-phase fraction in women with breast cancer. However, the PME relative peak area of spectra acquired *in vivo* from lactating breast is also significantly greater than that of non-lactating premenopausal breast (Twelves *et al.*, 1993). Indeed, the PME relative peak areas of lactating breast and cancers in post-menopausal women are similar (16.8% and 12.8% respectively; $P = 0.3$). There are several possible reasons why the PME signal should be increased under these two distinct circumstances. Firstly, PME may simply reflect the increased proportion of epithelial tissue, either normal or malignant, relative to fat and connective tissue in lactating breast or in the presence of a tumour. Alternatively, the elevated PME relative peak area may be due to increased membrane synthesis in hyperplastic or proliferating breast tissue. It is clear, however, that while an increased PME relative peak area is characteristic of malignancy this is not a specific feature and cannot be considered diagnostic of malignancy. The overlap between the PME relative peak areas of healthy and malignant breast, and the relatively low sensitivity of $^{31}$P-MRS *in vivo*, emphasise that attempts to use this technique to differentiate between normal and malignant breast tissue are unlikely to be successful.

Other apparent differences were detected between the tumours and healthy post-menopausal breast. The PCr relative peak area was higher in the tumours than in normal breast. This is in contrast to the findings *in vivo* of Sijens *et al.* (1988) and Redmond *et al.* (1991), and of Merchant *et al.* (1988), who studied human breast tumour extracts; all reported less PCr in breast tumours than in normal breast. However, the PCr relative peak area of healthy breast (Twelves *et al.*, 1993) and breast tumours is correlated with the CCW distance. In the current study 15 of the 19 patients were examined supine, whereas the spectra from healthy post-menopausal breast were acquired with the volunteer prone and the breast unsupported in order to reduce contamination by signal from the underlying chest wall. Therefore the apparently greater PCr signal from the tumours may reflect a shorter CCW distance in patients with tumours compared with those acting as healthy controls. Although a small PCr peak has been detected in PCA extracts of human breast cancer biopsies (Merchant *et al.*, 1988; Lowry *et al.*, 1992) and some breast cancer cell lines (Cohen *et al.*, 1986) it is likely that the main component of the PCr detected from breast tumours *in vivo* is contamination from the underlying chest wall muscle. Conclusions regarding the bioenergetics of breast tumours *in vivo* should not, therefore, be drawn from the PCr peak area or PCr/Pi peak area ratio.

Similarly, when expressed relative to the total $^{31}$P peak area of the spectrum the NTP relative peak area was significantly lower in the tumours than in normal breast. Although this might indicate areas of ischaemia or enhanced aerobic glycolysis within breast tumours, this difference was no longer apparent when PME was excluded from total $^{31}$P peak area. This indicates that these differences in NTP relative peak area were largely, if not entirely, secondary to alterations in the PME relative peak area and not a primary metabolic feature of the tumours.

Changes in the PME peak have also been a feature of human breast tumours studied *in vivo* from a total of ten patients following radiotherapy (Sijens *et al.*, 1988; Glaholm *et al.*, 1989), chemotherapy (Redmond *et al.*, 1992) and radiotherapy combined with chemotherapy (Ng *et al.*, 1989). Interestingly, all of the patients described previously had responded to treatment so the specificity of changes in $^{31}$P metabolites could not be evaluated. In the four women who received chemotherapy alone, a variety of changes in peak areas, including a fall in PME peak area ratio, were noted at differing times following treatment (Redmond *et al.*, 1992). In the current study spectroscopy was repeated at around 3 weeks, when the second cycle of many chemotherapy regimens is due but it is too early to evaluate response clinically. These data have confirmed that a fall in PME relative peak area is frequently seen in patients prior to changes in tumour size measured clinically in women who respond to treatment. However, one patient who later achieved a response did not have an early fall in PME relative peak area, while the patient with NC had shown a fall in PME relative peak area. Although Redmond *et al.* (1992) described a rise in the Pi/PME peak area ratio as characteristic of a response to treatment, in the current study Pi/PME fell significantly in one of the patients who responded. Changes in the PME relative peak area are not, therefore, uniformly predictive of response.

A frequent feature of animal MRS studies *in vivo* has been an increase in the high-energy phosphates of tumours following treatment. For example, Evanochko *et al.* (1983) described an increase in the PCr peak and decrease in the Pi peak from the 16/C mammary tumour following treatment with doxorubicin. These changes preceded alterations in tumour size. Sijens *et al.* (1988) described an increase in the PCr peak from two human breast cancers studied *in vivo* before and after radiotherapy. In the current study an increase in the PCr relative peak area was seen in all five patients who later responded but not in the patient who failed to respond. The CCW distance did not differ significantly between the serial spectroscopy studies. Nevertheless, since the PCr signal probably originates largely in the chest wall rather than the tumour or healthy breast, the rise in PCr relative peak area may represent increased contamination as a result of subtle changes in tumour size or composition. If changes in PCr relative peak area do reflect altered tumour size rather than metabolism, imaging techniques supplementing clinical measurement are likely to be more useful at present than MRS in monitoring early signs of response.

In conclusion, the current study has established that in post-menopausal women there are significant differences between the $^{31}$P-MR spectra of normal breast and carcinomas. Although the subjects were positioned differently for the acquisitions from healthy breast and tumours, the PME relative peak area of breast carcinomas was significantly higher than that of normal breast. However, a high PME relative peak area is also seen in healthy lactating breast and is not a specific feature of malignancy. This has important implications for the interpretation of the PME peak, which is increased in many types of tumour. The differences between tumours and normal breast with regard to the NTP relative peak area are secondary to the altered PME peak area, and variation in PCr is probably due to contamination. Therefore, these features do not reflect differences in tumour bioenergetics. Similarly, although an elevated PCr relative peak area was characteristic of an early response to chemotherapy, this may reflect slight physical rather than metabolic changes in the tumour. A fall in the PME relative peak area is frequently indicative of a response to chemotherapy, but at present this finding may not be sufficiently specific to be of use clinically. Further systematic studies are required in well-defined groups of patients, preferably using effective volume selection techniques such as conformal image-selected *in vivo* spectroscopy (ISIS) (Sharp & Leach, 1989) with absolute quantification of metabolites in order to define fully the role of $^{31}$P-MRS as both a research tool and a clinical investigation in patients with breast cancer.

### References

ACKERMAN, J.H., GROVE, T.H., WONG, G.G., GADIAN, D.G. & RADDA, G.K. (1980). Mapping of metabolites in whole animals by $^{31}$P NMR using surface coils. *Nature*, **283**, 167–170.

COHEN, J.S., LYON, R.C., CHEN, C., FAUSTINO, J., BATIST, G., SHOEMAKER, M., RUBALCABA, E. & COWAN, K.H. (1986). Differences in phosphate metabolite levels in drug-sensitive and -resistant human breast cancer cell lines determined by 31P magnetic resonance. *Cancer Res.*, **46**, 4087–4090.

DEGANI, H., HOROWITZ, A. & ITZCHAK, Y. (1986). Breast tumours: evaluation with P-31 MR spectroscopy. *Radiology*, **161**, 53–56.

EVELHOCH, J.L., KELLER, N.A. & CORBETT, T.H. (1987). Response-specific adriamycin sensitivity marker provided by *in vivo* 31P nuclear magnetic resonance spectroscopy in murine mammary adenocarcinoma. *Cancer Res.*, **47**, 3396–3401.

EVANOCHKO, W.T., NG, T.C., LILLY, M.B., LAWSON, A.J., CORBETT, T.H., DURANT, J.R. & GLICKSON, J.D. (1983). *In vivo* 31P NMR study of the metabolism of murine mammary 16/C adenocarcinoma and its response to chemotherapy, X-irradiation and hyperthermia. *Proc. Natl Acad. Sci. USA*, **80**, 334–338.

GLAHOLM, J., LEACH, M.O., COLLINS, D.J., MANSI, J., SHAR, J.C., MADDEN, A., SMITH, I.E. & MCCREADY, V.R. (1989). *In vivo* 31P magnetic resonance spectroscopy for monitoring treatment response in breast cancer. *Lancet*, **i**, 1326–1327.

HAYWARD, J.L., CARBONE, P.P., HEUSON, J.C., KUMAOKA, S., SEGALOFF, A. & RUBENS, R.D. (1977). Assessment of response to therapy in advanced breast cancer. *Eur. J. Cancer*, **13**, 89–94.

HOULT, D.I., BUSBY, S.J.W., GADIAN, D.G., RADDA, G.K., RICHARDS, R.E. & SEELEY, P.J. (1974). Observation of tissue metabolites using 31-P nuclear magnetic resonance. *Nature*, **252**, 285–287.

KALRA, R., WADE, K.E., HANDS, L., STYLES, P., CAMLEJOHN, R., GREENHALL, R., ADAMS, G.E., HARRIS, A.L. & RADDA, G.K. (1993). Phosphomonoester is associated with proliferation in human breast cancer: a $^{31}$P-MRS study. *Br. J. Cancer*, **67**, 1145–1153.

LENKINSKI, R.E., ALLMAN, T., SCHEINER, J.D. & DEMING, S.N. (1989). An automated iterative algorithm for the quantitative analysis of *in vivo* spectra based on the simplex optimisation method. *Magn. Reson. Med.*, **10**, 338–348.

LOWRY, M., PORTER, D.A., TWELVES, C.J., HEASLEY, P.E., SMITH, M.A. & RICHARDS, M.A. (1992). Visibility of phospholipids in $^{31}$P NMR spectra of human breast tumours *in vivo*. *NMR Biomed.*, **5**, 37–42.

MERCHANT, T.E., GIERKE, L.W., MENESES, P. & GLONEK, T. (1988). $^{31}$P Magnetic resonance spectroscopic profiles of neoplastic human breast tissues. *Cancer Res.*, **48**, 5112–5118.

MERCHANT, T.E., MENESES, P., GIERKE, L.W., DEN OTTER, W. & GLONEK, T. (1991). $^{31}$P Magnetic resonance phospholipid profiles of neoplastic human breast tissues. *Br. J. Cancer*, **63**, 693–698.

NG, T.C., GRUNDFEST, S., VIJAYAKUMAR, S., BALDWIN, N.J., MAJORS, A.W., KARALIS, I., MEANEY, T.F., SHIN, K.H., THOMAS, F.J. & TUBBS, R. (1989). Therapeutic response of breast carcinoma monitored by 31P MRS *in situ*. *Magn. Reson. Med.*, **10**, 125–134.

OBERHAENSLI, R.D., BORE, J., RAMLING, R., HILTON-JONES, D., HANDS, L. & RADDA, G.K. (1986). Biochemical investigation of human tumours *in vivo* with phosphorus-31 magnetic resonance spectroscopy. *Lancet*, **ii**, 8–11.

REDMOND, O.M., STACK, J.P., O'CONNOR, N.G., CODD, M.B. & ENNIS, J.T. (1991). *In vivo* phosphorus-31 magnetic resonance spectroscopy of normal and pathological breast tissues. *Br. J. Radiol.*, **64**, 210–216.

REDMOND, O.M., STACK, J.P., O'CONNOR, N.G., CARNEY, D.N., DERVAN, P.A., HURSON, B.J. & ENNIS, J.T. (1992). 31P MRS as an early prognostic indicator or patient response to chemotherapy. *Magn. Reson. Med.*, **25**, 30–44.

RUIZ-CABELLO, J. & COHEN, J.S. (1992). Phospholipid metabolites as indicators of cancer cell function. *NMR Biomed.*, **5**, 226–233.

SIJENS, P.E., WIJRDEMAN, H.K., MOERLAND, M.A., BAKKER, C.J.G., VERMEULAEN, J.W.A.H. & LUYTEN, R. (1988). Human breast cancer *in vivo*: H-1 and P-31 MR spectroscopy at 1.5 T. *Radiology*, **169**, 615–620.

SHARP, J.C. & LEACH, M.O. (1989). Conformal NMR spectroscopy: accurate localisation to noncuboidal volumes with optimal S.N.R. *Magn. Reson. Med.*, **11**, 376–388.

SMITH, T.A.D., GLAHOLM, J., LEACH, M.O., MACHIN, L., COLLINS, D.J. & AYNE, G.S. (1991). A comparison of *in vivo* and *in vitro* 31P NMR spectra from human breast tumours: variations in phospholipid metabolism. *Br. J. Cancer*, **63**, 514–516.

TWELVES, C.J., LOWRY, M., PORTER, D.A., DOBBS, N.A., GRAVES, P.E., SMITH, M.A. & RICHARDS, M.A. (1993). 31-phosphorus metabolism of human breast – an *in vivo* MRS study at 1.5 Tesla. *Br. J. Radiol.*, **67**, 36–45.